# 간호 대학생을 위한 전문직간 약물관련 교육프로그램의 연구동향: 주제범위 문헌고찰

**DOI:** 10.7586/jkbns.24.007

**Published:** 2024-05-28

**Authors:** Chaeyoon Jung, Jaeuk Oh, Sang Hui Chu

**Affiliations:** 1 College of Nursing, Yonsei University, Seoul, Korea; 1 연세대학교 간호대학; 2 Mo-Im Kim Nursing Research Institute, College of Nursing, Yonsei University, Seoul, Korea; 2 김모임 간호학 연구소

**Keywords:** Interprofessional education, Medication errors, Students, Nursing, 전문직간 교육, 약물오류, 간호학생

## Abstract

**Purpose:**

This study investigated how interprofessional education (IPE) related to medication for undergraduate healthcare students, including nursing students, has been designed and conducted. Furthermore, the outcomes of these IPE programs were evaluated.

**Methods:**

A scoping review was performed to understand the content and methods of IPE on medication for undergraduate healthcare students, including nursing students. A literature search was done using PubMed, Cumulative Index to Nursing and Allied Health Literature, Embase, Research Information Sharing Service, and Korean Nursing Database in English between January 2000 and December 2022. In total, 14 papers were selected and reviewed.

**Results:**

Eleven research studies were conducted on the implementation of one-time IPE on medication safety or errors. Among them, six studies focused on providing IPE to nursing students alongside medical and pharmacy students, while three studies targeted nursing and medical students, and two studies focused on nursing and pharmacy students. The most commonly employed educational method was simulation, which was utilized in 12 studies. All studies consistently reported a positive impact on attitudes toward IPE and effective communication. Additionally, five studies evaluating medication knowledge reported improvements in participants' understanding of drugs.

**Conclusion:**

The effects of IPE related to medication for undergraduate nursing students have been confirmed. It is necessary to introduce IPE in the field of pharmacology.

## 서론

### 1. 연구의 필요성

약물오류(medication error)는 약물의 처방(prescribing), 조제(dispensing), 투약(administrating), 모니터링(monitoring) 등 환자에게 약물을 사용하는 전 과정에서 발생하는 오류로 2022년 국내 환자안전사고 중 1위(43.3%)를 차지하고 있다[1]. 약물오류가 발생하게 되면 추가검사, 재조제, 보존적 치료 등으로 인해 환자의 입원기간이 연장되고 진료비가 상승할 뿐 아니라 심각한 경우 환자에게 영구적인 손상이나 사망까지 초래하게 된다[2]. 간호사는 업무시간 중 약 40%를 약물투약과 관련된 일을 하기에[3], 간호사의 약물 관련 지식, 비판적 사고, 임상추론 능력은 약물을 안전하게 투여하고 환자안전사고를 예방하는데 핵심적인 역할을 한다[4]. 그러나 약물오류는 의료인의 지식부족, 피로와 같은 인적 요인 뿐 아니라 조직 문화, 의사소통 등과 같은 인적자원 사이의 상호작용에 의해서도 발생하게 된다[5]. 약물오류의 원인을 조사한 Kim 등[3]의 연구에서도 약물오류의 원인으로 ‘구두 처방을 전달하는 과정에서의 의사소통의 오류에 의함’이 38.2%, ‘의료진들간의 미흡한 의사소통에 의함’이 34.1%로 약물 지식 외에도 의료진 사이의 의사소통 오류나 부족이 약물오류의 주요 원인으로 보고되고 있다. 따라서 약물오류를 예방하기 위해서는 처방, 조제, 관리, 투약과 모니터링 등 약물 투약 전 과정에 참여하는 의사, 간호사, 약사 등 여러 전문직이 서로 소통하고 협력하는 것이 필요하다.

최근 의료분야에서는 환자 중심의 의료제공이 강조됨에 따라 여러 분야의 의료 전문가들이 다학제적으로 환자에게 접근하는 전문직간 협력(interprofessional collaboration)이 강조되고 있다[6]. 점점 세분화되고 복잡해지는 의료환경에서 환자에게 안전하고 최적화된 의료서비스를 제공할 수 있는 전문직간 협력을 위해서는 둘 이상의 전문가들이 함께 배우는 전문직간 교육(interprofessional education)이 필요하다[7]. 전문직간 교육은 함께 배우는 과정에서 서로 다른 전문직의 역할과 책임, 각자의 전문직 정체성을 잘 이해하고, 팀워크, 의사소통과 같은 협업능력을 향상시키는 것으로 알려져 있다[8]. 유럽, 미국, 캐나다 등의 서구 국가에서는 전문직간 교육을 학부과정에 적용하여 전문직간 협력을 증진하고 환자안전사고를 예방하기 위해 노력하고 있으며[9], 최근에는 서로의 전문직에 대한 편견이나 고정관념이 자리잡기 전인 1학년부터 전문직간 교육을 도입하는 것이 효과적이라는 연구결과들이 제시되고 있다[10].

국내에서도 학부생을 대상으로 전문직간 교육을 시도하는 연구가 의학교육을 중심으로 증가하고 있으나 아직은 특정 질환에 대한 이해 또는 응급상황 시나리오를 주제로 제한적으로 이루어지고 있다[11-13]. 간호교육에서도 전문직간 교육의 중요성이 대두되면서 전문직간 교육의 필요성을 재고하는 연구들이 발표되고 있으나[6,14], 국내 교육기관에서 약물 또는 약물오류와 관련된 전문직간 교육을 도입한 사례와 그 효과에 대한 선행연구는 부족하다[15]. 또한 전문직간 교육이 환자안전 사고의 발생을 유의미하게 감소시킴에도 불구하고, 약물오류를 성과지표로 측정한 전문직간 교육에 관한 선행연구 대부분은 국외에서도 의사, 간호사, 약사 등 전문직을 대상으로 이루어지고 있는 실정이다[16].

국내에서 발생하는 약물사고가 처방(70.7%), 투약(21.4%), 조제(5.1%)의 단계에서 발생하고 있음을 고려해볼 때[1], 대부분의 투약을 담당하고 있는 간호사의 역할, 특히 간호사의 약물에 대한 지식과 의사소통 능력은 매우 중요하다. 간호대학 학부교과과정에는 간호술기(투약), 약물이 작용하는 원리와 약물이상 반응에 대한 이해(약리학), 약물적 치료를 포함하고 있는 간호학 전공과목 및 임상실습 등 다양한 약물관련 교과목이 존재하기 때문에 약물관련 전문직간 교육을 학부교과과정에 도입하고, 그 효과를 평가할 필요가 있다. 따라서, 본 연구에서는 간호대학생이 포함된 보건의료계열 학부생 대상의 약물 관련 전문직간 교육 프로그램에 대한 주제범위 문헌고찰을 시행하여 교육 프로그램의 특성을 분석하고 향후 간호대학생을 위한 약물 관련 전문직간 교육 프로그램 개발을 위한 기초자료를 제시하고자 한다.

## 연구 방법

### 1. 연구 설계

본 연구는 간호대학생이 포함된 보건의료계열 학부생을 대상으로 진행된 약물 관련 전문직간 교육의 내용과 방법 등을 파악하기 위한 주제범위 문헌 고찰(scoping review) 연구이다.

### 2. 연구 대상 및 자료 수집

주제범위 문헌고찰은 특정질문에 대한 상세한 답변이 아니라 광범위하게 개요를 확인하는 방법으로, 연구결과에 대한 상세한 보고보다는 연구의 결과를 요약하고 연구들을 개괄적으로 확인하여 이를 확산시키거나 연구간의 차이를 확인하는 것에 목적을 둔다[17]. 본 주제범위 문헌고찰은 Arksey and O’Malley [18]가 제시한 방법에 따라 진행되었다.

#### 1 단계: 연구질문 설정(identifying the research question)

주제범위 문헌고찰에서 연구질문은 연구에 대한 넓은 이해를 위해 폭넓은 접근의 연구질문을 설정할 것이 권고된다. 본 연구의 핵심질문은 다음과 같다.

1) 간호대학생이 포함된 보건의료계열 학부생들을 대상으로 한 약물 관련 내용을 포함한 전문직간 교육에 대한 연구의 일반적인 특징은 어떠한가?

2) 약물 관련 전문직간 교육의 구성(대상, 내용과 방법, 약물 종류)은 어떠한가?

3) 약물 관련 전문직간 교육의 효과는 어떠한가?

#### 2 단계: 관련 연구 검색(identifying relevant studies)

##### 1) 검색전략

본 연구는 보건의료계열 학생들을 위한 전문직간 교육과 관련된 논문이 수록된 6개의 국내외 데이터 베이스를 선정하여 검색하였다. 자료검색은 2023년 1월 15일부터 2023년 1월 19일까지 실시하였고, 출판 연도는 2000년 이후로 제한을 두어, 2022년 12월까지 해당 학술지에 출간된 논문 중 영어 또는 한국어로 출판된 실험논문을 대상으로 검토하였다. 국내외문헌을 검색하기 위해 PubMed, Cumulative Index to Nursing and Allied Health Literature (CINAHL), Embase, Cochrane, 학술연구정보서비스(Research Information Sharing Service, RISS), 한국 간호학 논문 데이터베이스(Korean Nursing Database, KNBASE)의 검색엔진을 활용했다. 전문직간 내용에 대한 검색 용어는 "Interprofessional" OR (multidisciplinary) OR "collaborative" OR ("interprofessionaleducation"[MeSHTerms]) OR ("interprofessionalrelations"[MeSHTerms]), 교육과 관련된 용어는 "learning" OR "education" OR "approach" OR "teaching", 약물과 관련된 검색용어는 (medicine) OR (medication) OR (drug) OR (pharmacology) OR ("error") OR ("safety") OR ("medicationerrors"[MeSHTerms]) OR ("InappropriatePrescribing"[MeSHTerms]), 학부 학생과 관련된 용어는(undergraduatestudent) OR “bachelor”, 간호학과 관련된 검색용어는("Nursing"[MeSH])이다.

##### 2) 자료 선정 및 배제 기준

본 연구의 문헌 선정기준은 2000년 1월부터 2022년 12월까지 국내외 학술지에 게재된 논문으로 간호대학생, 약학대학생, 의과대학생 등의 보건의료계열의 대학생들에게 시행된 전문직간 교육의 내용과 그 결과를 포함한 모든 연구를 대상으로 하였다. 포함된 문헌의 선정기준은 1)보건의료계열 학부생을 대상으로 한 연구 중에서도 간호대학생들을 포함한 연구, 2)전문직간 교육을 주제로 한 연구, 3)약물과 관련된 내용을 다룬 연구, 4)실험연구, 5)한글 또는 영어로 출판된 연구이며, 학술대회 포스터 및 초록, 학위논문, 평론, 월간지 등은 제외하였다.

#### 3 단계: 문헌 선정(study selection)

**Figure 1.**
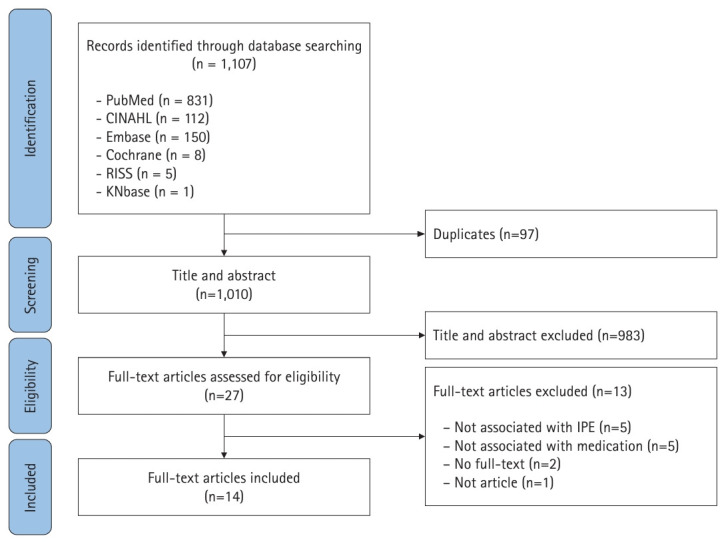
PRISMA flow chart for study selection.

PubMed, CINAHL, Embase, Cochrane, RISS, KNbase 검색엔진을 활용하여 검색하여 각각 831편, 112편,150편, 8편, 5편, 1편의 문헌이 도출되었다. 중복되는 문헌 97편을 제거하여 총 1,010편의 논문에 대하여 제목과 초록 수준에서 983편을 제외하였고 나머지 27편에 대해 두 명의 연구자가 1)간호대학생을 포함한 연구, 2)전문직간 교육을 주제로 한 연구, 3) 약물과 관련된 내용을 다룬 연구를 기준으로 판단하였다. 전문직간 교육과 관련된 주제가 아닌 5편의 논문, 약물과 관련되지 않은 5편의 논문, 전문이 존재하지 않는 2편의 논문, 서평 형식인 1편의 논문을 제외하였다(Figure 1). 전 과정은 2명의 독립적인 연구자가 수행한 후 결과를 비교하였으며, 검토결과가 일치하지 않은 경우 제3의 연구자가 개입하여 재검토하고 합의하는 과정을 거쳤다. 최종적으로 14편의 문헌을 선정하여 자료를 분석하였다(Appendix 1).

### 3. 자료 분석

#### 4,5 단계: 자료 기입, 분석, 요약 및 보고(charting, summarizing and reporting the results)

최종적으로 추출된 14편의 논문을 대상으로 논문 정보와 연구질문에 따른 핵심결과를 정리하기 위해 연구에서 사용한 자료분석틀에는 최종 선정논문의 저자 및 출판년도, 대상자 특성(전공, 학년, 성별, 수), 교육내용, 교육시간, 교육방식과 장소, 평가방법, 평가결과를 포함하였다. 수집된 자료는 분석틀에 따라 내용을 요약 정리하였으며 그 결과를 연구결과에 제시하였다.

## 연구 결과

### 1. 문헌의 일반적 특성

총 14편의 연구논문은 모두 2010년 이후 게재되었다. 국가별 논문 수는 미국에서 8편(57.1%), 영국에서 4편(33.3%), 뉴질랜드와 호주에서 각각 1편(8.3%)으로, 대부분 서구 국가에서 출간되었다.

연구 디자인의 경우, 14편 모두 약물 관련 전문직간 중재를 제공하였지만, 무작위화나 대조군을 설정하지 않았다. 14편 중 8편(57.1%)이 사전사후 평가를 하였으며, 나머지 6편(42.9%)은 사후 평가만을 실시하였다. 연구의 종류를 기준으로 분류하였을 때, 총 14편 중 양적 연구만 다룬 논문은 6편(42.9%), 양적 연구 결과와 함께 개방형 질문을 통해 주관식으로 평가한 연구는 8편(57.1%)이었다. 표본수의 경우, 8명에서 440명으로 다양하였으며, 14편 논문의 표본 수 평균은 147.5명이었다(Table 1).

### 2. 전문직간 교육 프로그램의 특성

#### 1) 대상자

약물 관련 전문직간 교육 프로그램에 참여한 학습자는 간호대학생, 약학대학생, 의과대학생이 참여한 논문이 6편(42.9%)으로 가장 많았고, 간호대학생과 의과대학생이 4편(28.6%), 간호대학생과 약학대학생이 3편(21.4%)이었으며, 간호대학생, 약학대학생, 의과대학생과 영양학과학생이 참여한 논문이 1편(7.1%)이었다.

학년별 분포를 살펴보았을 때, 학년을 보고하지 않은 5편의 논문을 제외한 9편의 논문에서 간호대학생의 학년 분포는 4학년이 7편(77.8%)으로 가장 많았고, 3학년, 2학년이 각각 1편(11.1%)이었다. 의과대학생의 경우 4학년이 7편(70.0%), 3학년이 1편(10.0%), 2학년이 2편(20.0%), 약학대학생의 경우 4학년이 5편(55.6%), 3학년, 2학년이 각각 2편(22.2%), 영양학과 학생의 경우 4학년이 1편(100.0%)으로 대부분의 논문에서 4학년 학부생을 대상으로 전문직간 교육을 시행하였다(Table 1).

#### 2) 교육 프로그램의 내용과 방법

14편의 논문의 내용은 약물 안전(medication safety)과 약물 관련 지식을 주제로 한 논문으로 나눌 수 있었다. 약물 안전을 주제로 한 연구는 총 13편(92.85%), 약물 관련 지식을 주제로 하는 연구는 1편(7.14%)이었다. 또한 약물 안전의 하위주제로 약물 오류를 다룬 연구는 6편이었다(Table 2).

각 연구에서는 한 팀당 최소 3명, 최대 12명의 서로 다른 전문직 학생들로 팀을 구성하여 교육을 제공하였다. 교육 운영 장소로는 별도의 시뮬레이션 실험실에서 진행한 경우가 8편(57.1%)으로 가장 많았고 강의실에서 운영한 경우가 3편(21.4%), 병원에서 진행한 경우가 2편(14.3%), 장소를 밝히지 않은 경우가 1편(7.1%) 이었다. 각 논문에서 기술된 주 교육 방식은 시뮬레이션이 12편(85.7%%), 강의를 제공한 논문이 2편(14.3%)이었다. 이외에도 추가적으로 학생들끼리 논의할 수 있는 시간을 제공한 논문이 11편 (78.6%), 디브리핑할 수 있는 시간을 제공한 논문이 8편(57.1%) 이었다. 교육의 운영기간은 14편중 10편(71.4%)의 연구에서 일회성으로 교육을 제공하였으며 이중 1편을 제외한 모든 논문에서 시뮬레이션을 교육방식으로 활용하였고 시뮬레이션은 한 번에 최소 7분에서 최대 50분 동안 제공되었다(Table 3).

#### 3) 교육에 활용된 약물

고찰한 논문 중 교육에 가장 빈번하게 활용된 약물은 항응고제(dabigatran 등)으로 총 3편(21.4%)의 논문에서 활용되었다. 항생제(penicillin, tazobactam 등), 파킨슨병(Parkinson’s disease) 치료제는 각각 2편(14.3%)의 논문에서 활용하였으며, 해열진통제(ibuprofen, paracetamol 등), 기관지확장제(aminophylline), 인슐린제제(novorapid 등), 정신질환제(lithium 등), 심혈관제제(digoxin 등)를 각각 한 편(7.1%)의 논문에서 교육에 활용하였다(Table 2).[Table t3-jkbns-24-007]

### 3. 전문직간 교육 프로그램의 효과 평가

전문직간 교육 프로그램의 효과는 약물관련 지식 향상과 전문직간 교육 경험에 대한 인식 및 태도 변화에 대해 평가하였다. 14편의 논문 중 3편(21.4%)은 약물과 관련된 지식과 전문직간 교육 경험에 대한 인식 및 태도를 동시에 평가하였으나[A4,A7,A12], 9편(64.3%)은 전문직간 교육 프로그램 경험에 대한 인식만을 평가하였고[A1,A3,A5,A6,A8,A9,A11,A13,A14] 나머지 2편(14.3%)에서는 약물과 관련된 지식만을 평가하였다[A2,A10].

약물과 관련된 지식을 평가한 5편의 논문 중2편(40.0%)은 지식 향상에 대한 학생들의 주관적인 인식을 설문 조사하였고[A7,A12], 퀴즈 등의 교육 중재 전후의 시험 점수를 이용하여 객관적으로 지식의 향상을 평가한 논문은 3편 (60.0%)이었다[A2,A4,A10]. 5편 논문 모두에서 전문직간 교육 중재 후 학생들의 주관적인 약물 지식, 객관적인 약물 퀴즈 점수가 유의미하게 향상되었다.

중재 전후의 전문직간 교육에 대한 인식 및 태도를 평가한 12편의 사용된 도구는 논문마다 매우 다양했는데, Readiness for Interprofessional Learning Scale을 사용한 논문이 3편(21.4%)[A5,A9,A13], Attitude Toward Healthcare Teams Scale을 사용한 논문, Teamwork Attitudes Questionnaire을 사용한 논문이 각각 1편(7.1%)이었으며[A7,A8], 이외에는 자체적으로 설문 도구를 개발하여 사용하였다. 다양한 측정도구를 사용했음에도, 전문직간 교육 프로그램 제공 전, 후 대부분의 지표에서 통계적으로 유의미한 차이를 확인했다(Table 4).

주관식 평가의 경우, 8편의 논문 중 만족도를 평가한 논문이 4편(50.0%)으로 가장 많았으며[A1,A3,A5,A9], 학습 경험을 평가한 논문이 3편(37.5%)[A6,A12,A14], 약리학 지식과 함께 전문직간 역할을 확인한 논문이 1편(12.5%)이었다[A7]. 주관식 평가를 진행한 8편의 논문 모두에서 공통적으로 보건의료계열 전문직 각자의 역할뿐만 아니라 상호작용을 통해 서로의 역할에 대한 인식을 높일 수 있었다는 것이 보고되었다. 특히, 질적 평가결과만을 보고한 연구에서 간호, 의과대학생은 교육내용을 임상에서 적용할 수 있을 것이라고 답했으며, 약학대학생은 임상에서의 협력을 위해 약물 관련 전문직간 교육 프로그램이 더 많이 시행되어야 한다고 언급하였다[A5].

## 논의

최근 의료분야에서는 환자 중심의 의료제공이 강조됨에 따라 의료기관내 다양한 전문직이 서로 협력하여 대상자에게 의료서비스를 제공하는 전문직간 협력의 중요성이 대두되고 있다[6]. 본 연구는 간호대학생을 대상으로 약물 관련 전문직간 교육 프로그램을 제공한 14편의 논문을 고찰하여, 향후 간호대학생을 위한 약물 관련 전문직간 교육개발에 필요한 기초자료를 제공하고자 시행되었다.

고찰한 14편의 연구 중 약물과 관련된 지식 자체의 향상만을 평가한 연구는 2편(14.29%)뿐인 반면, 전문직간 교육에 대한 태도나 의사소통과 같은 협업수준을 평가한 연구는 9편(64.29%), 지식의 향상과 태도, 협업수준을 함께 평가한 연구는 3편(21.43%)이었다. 이를 통해 전문직간 교육의 목적이 학생들의 팀워크, 의사소통, 리더십과 같은 협업능력을 극대화하는 것에 초점을 맞춰온 것처럼[19], 약물관련 전문직간 교육 역시 약물에 대한 지식 향상보다는 약물오류의 주된 원인으로 알려진 협업 및 의사소통 능력 함양을 목적으로 한다는 것을 알 수 있었다. 안전한 투약을 가능하게 하는 요인으로 의사소통의 중요성을 강조한 Fortescue 등[20]의 연구결과에 따르면 약물 오류 중 47%는 의사와 약사의 의사소통의 개선을 통해, 17%는 의사와 간호사 간의 의사소통의 개선을 통해 해결될 수 있다고 한다. 따라서, 약물 오류를 예방하고 최소화하기 위해서는 효과적인 팀워크와 의사소통능력 함양을 학습과제로 하는 학부과정의 약물관련 전문직간 교육이 필요하다[21]. 한편,전문직간 교육 후, 약물 관련 지식 수준의 향상을 평가한 5편의 연구에서는 5편 모두 학생들의 주관적, 객관적 지식 수준이 유의미하게 향상되었다. 특히, 임상실습 경험을 바탕으로 졸업 학년의 약학대학생이 간호대학생에게 9주간 다양한 약물치료에 대한 강의를 진행한 연구에서 간호대학생의 약물관련 지식이 유의하게 향상된 결과는 또래를 통한 배움이 약리학 과목에서 효과적인 학습방법이 될 수 있음을 시사하기에[A10], 2학년에 주로 수강되는 약리학 교과목에 도입을 고려해 볼만한 교육방법이다.

14편의 연구 대부분이 각 전문직(간호,의학, 약학 등)의 학부 마지막 학년(4학년)을 대상으로 교육을 진행하였다. 이는 고학년 학생일수록 학부과정을 통해 축적된 지식과 자신의 전공 정체성 및 역할에 대한 확고한 이해를 바탕으로 전문직간 교육의 효과를 극대화할 수 있기 때문인 것으로 볼 수 있다[22]. 그러나, 보건의료계열 학생들은 대학 입학과 동시에 직업적 가치를 내면화하고 전문직 사회화 과정을 경험하면서 다른 전공에 대한 고정관념을 가지게 되므로[23], 고학년보다는 저학년에서부터 전문직간 교육을 제공하는 것이 더욱 효과적이라는 연구가 증가하고 있으므로[24,25] 약리학 과목에 전문직간 교육을 도입하는 것을 심도있게 고려할 필요가 있다. 일본의 경우에도 41%의 대학에서 의예과 1학년때부터 전문직간 교육을 시행하고 있으며 본과 3학년 이상에서는 11%만이 전문직간 교육을 시행하고 있다[26].

총 14편의 논문 중 전문직간 교육방법으로 가장 많이 활용한 방법은 학생들이 스스로 협업하며 문제해결에 참여할 수 있는 시뮬레이션이었다. 시뮬레이션 기반 학습을 통해 학생들은 안전한 환경에서 현실감 높은 환자 시나리오를 통해 임상 기술을 연습하고 자신감을 형성하며, 비판적 사고와 의사결정 기술을 향상시킬 수 있다[27]. 시뮬레이션을 이용한 교육은 학생들에게 현실감 넘치는 가상의 의료상황을 간접적으로 경험하게 해, 투약오류를 줄이는 데에 일반 교육보다 효과적이라고 보고되었으며[28], Institute of Medicine에서도 투약 오류를 줄이기 위한 의료진 교육 방식에 시뮬레이션 교육을 권고한 바 있다[29]. 시뮬레이션을 전문직간 교육에 활용하는 것은 의사소통 기술, 협력, 팀워크를 향상시킬 뿐 아니라[29], 약물 지식을 오랫동안 기억하게 하고 임상추론을 도와 강의실에서 독립적으로 진행되는 약리학 강의의 제한점을 극복하고 임상응용을 촉진하는 교수법으로 제안된 바 있다[30]. 시뮬레이션을 활용한 12편의 연구에서 모두 디브리핑, 피드백, 토의와 같은 상호 의견 교환의 단계를 거쳤는데, 학생들은 상호 의견 교환 단계를 통해 전문직간 교육 프로그램 중 자신이 수행했던 역할이나 행동에 대해 성찰하게 된다. 특히, 시뮬레이션 학습에서 디브리핑은 가장 핵심적인 단계로, 효과적인 시뮬레이션을 위해 가장 중요하다[31]. 디브리핑 과정을 통해 학생들은 시나리오 수업 후, 구조화된 성찰을 하고 이 과정을 통해 학습을 완성하게 되는데[32], 디브리핑은 학생들의 적극적인 참여, 성찰, 반복학습을 유도하여 학습에 대한 관심 몰입, 행동 변화 등을 일으키고[33], 임상 판단 능력과 비판적 사고 능력을 향상시킨다[34].

총 14편의 연구 중, 11편의 연구가 1회성 전문직간 교육에 그쳤는데, 이는 전문직간 교육의 유용성에도 불구하고, 각 전공별로 면허를 취득하기 위해 엄격하게 요구되는 교육과정, 전공별로 다른 시간표와 실습 운영 등으로 인해 새로운 교과목을 추가하거나 교육방법을 도입하는 등 유연한 교육과정을 운영하는데 어려움이 있기 때문이다[35]. 보건의료계열에서 효과적으로 전문직간 교육을 학부과정에 도입하기 위해서는 관련 전공 교수가 모두 참여하는 다학제적 위원회를 구성하여 전문직간 교육을 기관 차원에서 설계, 도입, 운영하려는 노력이 중요하다[36]. 따라서, 국내에서 전문직간 교육이 활성화되기 위해서는 우선 각 대학 내 보건의료계열 전공 교수 협의체 구성을 통해 학년별 역량과 성취수준을 고려한 단계적인 목표 설정이 필요할 것이다. 이러한 노력과 함께 1회성 교육이라도 학년별로 잘 설계된 교과/비교과 과정을 통하여 전문직간 교육을 주기적으로 도입한다면 학생들은 졸업 시까지 체계적으로 전문직간 교육을 경험할 수 있을 것이다.

약물관련 전문직간 교육의 효과는 주로 전문직간 교육에 대한 인식 및 태도의 변화를 주로 측정하였고, 일부 연구만 약물관련 지식 수준을 평가한 걸로 확인되었다. 반응, 학습, 행동, 결과의 4개 수준에서 평가하는 Kirkpatrick 평가모델은 교육과정을 단계적으로 설계하고 평가하는데 유용하다[37]. Kirkpatrick 평가모델을 확장시킨 Reeves 등[38]의 연구에서는 교육에 대한 평가수준을 학습자의 반응(1단계), 태도/인지의 수정(2a단계), 지식과 기술의 습득(2b단계), 행동적 변화(3단계), 조직적 실무의 변화(4a단계), 환자, 가족, 지역사회에의 이익(4b단계)로 세분화하였다. 전문직간 교육에 대한 만족도, 자신감, 팀구성과 리더십에 대한 생각, 상호간 지지와 의사소통, 자기효능감, 오류를 보고하는 것에 대한 개방적인 태도 등 본 연구를 통해 주로 확인된 평가는 1-2단계 수준이었다. 이는 대부분의 연구가 1회성 전문직간 교육에 그쳐 행동적 변화를 유발하기에는 부족하였기 때문이라고 생각된다. 따라서, 전문직간 교육을 통해 약물오류를 예방하기 위해서는 학부과정에서 협력과 의사소통에 대한 태도뿐만 아니라 투약관련 지식과 기술 습득, 시뮬레이션 환경하에서 행동적 변화를 이끌 수 있도록 설계되어야 할 것이다. 또한 졸업 후에도 임상현장에서 약물오류에 관한 전문직간 교육이 지속되어야 비로소 약물오류를 감소시켜 환자안전에 기여하는 역량 있는 간호사를 양성할 수 있을 것이다.

## 결론

본 주제범위 문헌 고찰 결과, 간호대학생이 포함된 보건의료계열 학부생들을 위한 약물과 관련된 전문직간 교육을 중재로 활용한 논문은 14편으로, 아직 연구가 제한적임을 확인하였다. 그러나, 대부분의 연구에서 1회성으로 전문직간 교육을 제공했음에도 불구하고 학생들의 전문직간 교육에 대한 인식과 태도가 개선되고, 약물 지식이 향상되었다는 긍정적인 결과를 보여주었다. 따라서 국내 간호교육 과정에서도 약리학 또는 임상실습 과목 등을 활용하여 약물을 주제로 전문직간 교육을 개발하고 도입할 필요가 있다.

## Figures and Tables

**Table 1.** Characteristics of Included Studies (N = 14)

	Author (yr)	Purpose	Research design	Sample size (n)	Learner characteristics (yr)
	Country				
[A1]	Achike et al. (2014)	To draw attention to the need for IPE in RDU by describing a pilot interprofessional class that could serve as base material for developing an IPE program for the RDU curriculum	Post-test only	108	Nursing (fourth year)
	USA		Quasi-experimental		Medicine (second year)
[A2]	Clay et al. (2017)	To determine if medical and nursing students can identify hazards of hospitalization that could result in harm to patients and to detect differences between professions in the types of hazards identified	Pre- post	144	Nursing (fourth year)
	USA		Quasi-experimental		Medicine (fourth year)
[A3]	Curley et al. (2019)	To assess pharmacy students’ opinions of an IPE course in their final year of the Bachelor of Pharmacy program at The University of Auckland	Post-test only	304	Nursing (fourth year)
	New Zealand		Quasi-experimental		Medicine (third year)
					Pharmacy (fourth year)
[A4]	Ellis et al. (2022)	To investigate the impact of an interprofessional mock code on students’ comfort and competency related to PD medication administration during care transitions	Pre-post	202	Nursing (fourth year)
	USA		Quasi-experimental		Medicine (fourth year)
[A5]	Hardisty et al. (2014)	To encourage IPE and work to promote patient-focused attitudes to medical use	Pre-post	318	Nursing (fourth year)
	UK		Quasi-experimental		Medicine (fourth year)
					Pharmacy (fourth year)
[A6]	Kayyali et al. (2019)	To adapt existing nursing simulation-based learningto include pharmacy content	Post-test only	440	Nursing (year n/r)
	UK		Quasi-experimental		Pharmacy (year n/r)
[A7]	Meyer et al. (2017)	To examine the feasibility of an interprofessional high-fidelity pharmacology simulation and its impact on pharmacy and nursing students’ perceptions of inter-professionalism and pharmacology knowledge	Pre-post	146	Nursing (year n/r)
	USA		Quasi-experimental		Pharmacy (second year)
[A8]	Motycka et al. (2018)	To utilize simulated medication management scenarios in interprofessional scenarios in an interprofessional training environment and to evaluate the impact of the training on team-based stills and attitudes	Pre-post	48	Nursing (second year)
	USA		Quasi-experimental		Medicine (fourth year)
					Pharmacy(second and third years)
[A9]	Paterson et al. (2015)	To develop, pilot, and test the feasibility of a simulated inter-professional prescribing master class for non-medical prescribing students, medical students, and pharmacists	Pre-post	8	Nursing (year n/r)
	UK		Quasi-experimental		Medicine (fourth year)
					Pharmacy (year n/r)
[A10]	Powell et al. (2020)	To compare student nurses’ knowledge base, self-confidence, and socialization to and value of interprofessional interaction at baseline and after nine weekly pharmacotherapy reviews from student pharmacists	Pre-post	25	Nursing (year n/r)
	USA		Quasi-experimental		Pharmacy (fourth year)
[A11]	Ragucci et al. (2016)	To evaluate the impact of an interprofessional communication skills workshop on pharmacy students’ confidence and proficiency in disclosing medical errors to patients	Post-test only	75	Nursing (fourth year)
	USA		Quasi-experimental		Medicine (second year)
					Pharmacy (third year)
[A12]	Schussel et al. (2019)	To measure the impact of an interprofessional experience in MTM on students’ attitudes and skills regarding interprofessional collaboration	Post-test only	27	Nursing (year n/r)
	USA		Quasi-experimental		Medicine (year n/r)
					Pharmacy (fourth year)
					Nutrition (fourth year)
[A13]	Stewart et al. (2010)	To develop and evaluate an interprofessional workshop focusing on pediatric drug prescribing and administration	Pre-post	172	Nursing (third year)
	UK		Quasi-experimental		Medicine (fourth year)
[A14]	Young et al. (2021)	To educate students about the role of interprofessional collaboration in medication safety	Post-test only	48	Nursing (fourth year)
	Australia		Quasi-experimental		Medicine (fourth year)
					Pharmacy (fourth year)

n/r = Not reported; IPE = Interprofessional education; RDU = Rational drug use; PD = Parkinson’s disease; MTM = Medical therapy management.

**Table 2.** Characteristics of Interprofessional Education Programs (N = 14)

Author (yr)	Topics	Themes	Setting	Teaching method	Duration of intervention (hours per day/case)
Country					
Achike et al. (2014)	Medication safety	Prescribing, administration, monitoring	Classroom	Lecture	1 time (2 hours/no case)
USA		- Case(s): cough, sore throat			
[A1]		- Medication(s): cough suppressant (dextromethorphan)			
Clay et al. (2017)	Medication safety, Medication errors	Prescribing, monitoring	Simulation center	Simulation	2 times (4 hours/1 case)
USA		- Case(s): medication allergy, medication with the wrong patient			
[A2]		- Medication(s): penicillin/tazobactam (allergy), metronidazole (medication with the wrong patient)			
Curley et al. (2019)	Medication safety, Medication errors	Prescribing	Simulation center	Simulation	2 times (4 hours/4 cases)
New Zealand		- Case(s): pain management, respiratory difficulty, anticoagulant overdose, neurological symptom, seizure			
[A3]		- Medication(s): anticoagulant, lithium			
Ellis et al. (2022)	Medication safety	Administration	Simulation center	Simulation	1 time (n/r)
USA		- Case(s): PD			
[A4]		- Medication(s): PD medication			
Hardisty et al. (2014)	Medication safety	Prescribing, administration, monitoring	Hospital	Simulation	1 time (6 hours/2–3 cases)
UK		- Case(s): shortness of breath and purulent sputum			
[A5]		- Medication(s): n/r			
Kayyali et al. (2019)	Medication safety	Administration, monitoring	Simulation center	Simulation	1 time (2.5 hours/1 case)
UK		- Case(s): stroke, COPD, pain management, head injury, hypertension, dementia, diabetes, PD			
[A6]		- Medication(s): n/r			
Meyer et al. (2017)	Medication safety	Prescribing, administration, monitoring	Simulation center	Simulation	1 time (1 hour/1 case)
USA		- Case(s): heart failure			
[A7]		- Medication(s): digoxin (toxicity)			
Motycka et al. (2018)	Medication safety, Medication errors	Prescribing, administration, monitoring	Simulation center	Simulation	1 time (4 hours/4 cases)
USA		- Case(s): patient with the wrong chart (wrong chart with the same name), medication allergy, medication toxicity			
[A8]		- Medication(s): penicillin (allergy)			
Paterson et al. (2015)	Medication safety	Prescribing, monitoring	Simulation center	Simulation	1 time (2.5 hours/3 cases)
UK		- Case(s): sepsis, diabetes, hypertension, adverse drug reaction			
[A9]		- Medication(s): n/r			
Powell et al. (2020)	Medication knowledge	Pharmacology education	Classroom	Lecture	9 times (weekly) (1.5 hours/no case)
USA		- Topic: pulmonary medication, antibiotics, cardiovascular medication, diabetes medication, antidepressants, pain management, antipsychotics, GERD, inflammatory bowel disease			
[A10]		- Medication(s): n/r			
Ragucci et al. (2016)	Medication safety, Medication errors	Monitoring	Simulation center	Lecture	1 time (2 hour/1 case)
USA		- Case(s): bleeding due to an antidepressant		Simulation	
[A11]		- Medication(s): anticoagulant			
Schussel et al. (2019)	Medication safety	Monitoring	Classroom	Simulation	3 times (weekly) (7 hours/2–3 cases)
USA		- Case(s): n/r			
[A12]		- Medication(s): n/r			
Stewart et al. (2010)	Medication safety, Medication errors	Prescribing, administration, monitoring	n/r	Simulation	1 time (2 hours/4 cases)
UK		- Case(s): upper respiratory tract infection, asthma			
[A13]		- Medication(s): ibuprofen, paracetamol, aminophylline			
Young et al. (2021)	Medication safety, Medication errors	Administration, monitoring	Hospital	Simulation	1 time (2 hours/1 case)
Australia		- Case(s): fracture, pneumonia, cellulitis, diabetes, pain management, schizophrenia			
[A14]		- Medication(s): anticoagulant (dabigatran), insulin (Novorapid)			

n/r = Not reported; PD = Parkinson’s disease; COPD = Chronic obstructive pulmonary disease; GERD = Gastroesophageal reflux disease.

**Table 3. t3-jkbns-24-007:** Details of Interprofessional Education Programs (N = 14)

Author (yr)	Program	Group allocation	Description of class procedure
Country			
Achike et al. (2014)	RDU through rational drug prescribing	Nursing (n = 20)	•Lecture (15 min)
USA		Medicine (n = 88)	- Students participated in a lecture about rational drug choice, the characteristics of a good prescription order, the 7 “rights” of medication administration
[A1]		10–12 people per group (2–3 nursing students were assigned to groups with 7–8 medical students)	•Group discussion (30 min)
			- With a shared scenario, each group had a discussion and wrote a prescription following World Health Organization-recommended prescription manual
			•Presentation (50 min)
			- Representatives of each group presented their groups’ process of drug choice and prescription
			•Feedback (20 min)
			- Students gave feedback on their individual experience of the program anonymously and voluntarily
			•Post-test questionnaire
Clay et al. (2017)	“Room of Horrors” simulations to identify patient safety hazards	Nursing (n = 51)	•Simulation as individuals (7 min)
USA		Medicine (n = 93)	- For general hospitalized patients, it included hazards specific to infection control, hospital-acquired infections, skin breakdown, and delirium
[A2]		3–4 people per group	•Simulation as a team (7 min)
			- With four features developed by faculty: identifying categories of hazards, an ICU-based simulation, inclusion of overlap, incorporation of the medical record
			•Group discussion
			•Half-day patient safety discussion, very short review from faculty
			•Post-test questionnaire
Curley et al. (2019)	“Ward Sim” IPE course	Nursing (year n/r)	•Classroom activity, day1
New Zealand		Medicine (year n/r)	- Information given about the ISBAR communication tool before the course
[A3]		Pharmacy (n = 96)	•Simulation, day1 (30 min)
		8–11 people per group	- Undifferentiated problems arising in inpatients that required assessment and management of the patient but not resuscitation.
			•Debriefing, day1 (30 min)
			•Speaking up activity, day2
			- Structured framework that included the use of a pneumonic for managing concerns (PACER)
			•Simulation, day2 (30 min)
			- Four cases included
			•Debriefing, day2 (30 min**)**
			•Post-test questionnaire
Ellis et al. (2022)	Interprofessional mock code focusing on students’ comfort and competency related to PD medication administration during care transitions	Nursing (n = 113)	•Pre-test questionnaire
USA		Medicine (n = 32)	•Simulation
[A4]		Pharmacy (n = 22)	- Mock code situation; acute care setting with a patient with PD in a care transition
			•Debriefing
			- Quality improvement strategies for safe and timely medication administration and reconciliation during the care transition
			•Post-test questionnaire
Hardisty et al. (2014)	Medication prescribing seminars	Nursing (n = 52)	•Pre-test questionnaire
UK		Medicine (n = 190)	•Seminar (2–3 hours)
[A5]		Pharmacy (n = 76)	- Five detailed scenarios, summaries of the 10 most prescribed drug classes, a series of case studies and group discussions covering prescribing skills
		5–6 people per group	•Post-test questionnaire
Kayyali et al. (2019)	Simulation in pharmacy education	Nursing (n = 314)	•Hospital setting session
UK		Pharmacy (n = 126)	•Introduction (15 min)
[A6]			•Briefing (15 min)
			•Scenario simulation (45 min)
			•3 debriefing time points (15 min, 15 min, 60 min)
			•Post-test questionnaire
			•General practice setting session
			•Introduction (15 min)
			•Briefing (15 min)
			•Scenario simulation (60 min)
			•Debriefing (15 min)
			•Post-test questionnaire
Meyer et al. (2017)	Interprofessional high-fidelity pharmacology simulation	Nursing (n = 64)	•Pre-test questionnaire
USA		Pharmacy (n = 79)	•Simulation (30 min)
[A7]		7–8 people per group (3–4 nursing students are assigned with 3–4 pharmacy students)	- Patient case with supporting documents, a simulation script involving digoxin toxicity and the need for vasopressor administration, and a debriefing guide
			•Debriefing (30 min)
			•Post-test questionnaire
Motycka et al. (2018)	Interprofessional medication management simulation	Nursing (n = 21)	•Pre-test questionnaire
USA		Medicine (n = 12)	•Simulation (10 min per each scenario, 4 scenarios in total)
[A8]		Pharmacy (n = 15)	- Rotating different medication management scenarios
		4 people per group (1 medicine, 1 pharmacy, and 2 nursing students)	•Debriefing (10 min per every scenario)
			•Post-test questionnaire
Paterson et al. (2015)	Simulated interprofessional prescribing master class	Nursing (n = 3)	•Pre-test questionnaire
UK		Medicine (n = 2)	•Simulation (45 min for each scenario, 3 scenarios in total)
[A9]		Pharmacy (n = 3)	- One sepsis, one polypharmacy and one community-based case
		3 people per group (1 medicine, 1 pharmacy, and 1 nursing student)	•Debriefing (after every scenario)
			•Post-test questionnaire
Powell et al. (2020)	Pharmacotherapy knowledge-based peer-teaching for nursing students by pharmacy students	Nursing (n = 25)	•Pre-test questionnaire
USA		Pharmacy (n = 4)	•Didactic lectures (9 weekly peer teaching education) and education discussion (60-90 min per each topic)
[A10]			- Basics of pathophysiology, the most commonly used medications to treat a specific disease, brand and generic names, mechanisms of action, side effects, drug administration, and common drug interactions related to specific educational topics
			• Post-test questionnaire
Ragucci et al. (2016)	Interprofessional communication skills workshop in disclosing medical errors to patients	Nursing (n = 18)	•Workshop (1-hour lecture)
USA		Medicine (n = 36)	- For the active intervention group, the participants received a lecture on the key steps in error disclosure
[A11]		Pharmacy (n = 75)	•Simulation (50 min)
		4 people per group	- The same case required students to care for an acute gastrointestinal bleed secondary to a medication error
		(1 medicine, 2 pharmacy, and 1 nursing student)	•Debriefing (10 min)
			•Post-test questionnaire
Schussel et al. (2019)	Interprofessional experience in medication therapy management	Nursing (n = 7)	•Pre-test questionnaire
USA		Medicine (n = 4)	•Interprofessional activity as a team of 6 members (2 hours)
[A12]		Pharmacy (n = 8)	- Complex case studies to practice teamwork and communication strategies.
		6 people per group (2 students from each profession)	•Constructive feedback from preceptors
			•Interprofessional activity as a team of 3 members (5 hours)
			- Preparing for and calling actual patients
			•Debriefing
			•Post-test questionnaire
Stewart et al. (2010)	Interprofessional approach to improving pediatric medication safety	Nursing (n = 21)	•Pre-test questionnaire
UK	(drug prescribing and administration)	Medicine (n = 48)	•Interprofessional workshop with “real-life” clinical scenarios (2 hours)
[A13]		2–3 people per group	- Required to prescribe the appropriate drug, calculate the correct dosage, accurately complete a Drug Kardex, prepare the drug for administration, identify alternative drugs where appropriate, and be able to provide information to parents.
			• Debriefing
			• Post-test questionnaire
Young et al. (2021)	Medication safety workshop	Nursing (year n/r)	• Pre-test questionnaire
Australia		Medicine (year n/r)	- Interprofessional medication safety workshop
[A14]		Pharmacy (year n/r)	• Introduction (5 min)
			• Rotate through 4 stations (20 minperstation, 1: simulated patient history, 2: bag of meds, 3: pop quiz, 4A– 4D: case studies)
			• Case presentations (15 min)
			• Closure (15 min)
			• Evaluation (5 min)
			• Post-test questionnaire

n/r = Not reported; RDU = Rational drug use; IPE = Interprofessional education; ISBAR = Introduction, situation, background, assessment, request/recommendation; PACER = Probe, alert, challenge, emergency, react; PD = Parkinson’s disease.

**Table 4.** Outcomes of Interprofessional Education Programs (N = 14)

Author (yr)	Variables	Measurement(s)	Outcome
Country			
Achike et al. (2014)	Perception of IPE experience (AD, AO)	6 items, 4-point Likert scale	•AD score: 0.6 ± 0.1 (n = 234) (*p* < .01)
USA		- 3 items for AD theme and 3 items for AO themes	•AO score: 1.0 ± 0.1 (n = 238) (*p* < .01)
[A1]		*Open-ended questions for satisfaction	•Overall aggregate score: 0.8 ± 0.1 (*p* < .01)
Clay et al. (2017)	Medication knowledge (medication/allergy-related hazards)	Frequency of hazards identified	•Interprofessional teams performed better than individuals in identifying medication administration errors (*p* < .05).
USA			
[A2]			
Curley et al. (2019)	Perception of IPE experience	8 items, 5-point Likert scale	•Nursing students agreed/strongly agreed that they learned strategies to improve the safety of prescribing medications (66.6%).
New Zealand		*Open-ended questions for satisfaction, and lessons learned	•Nursing students agreed/strongly agreed that learning with other professional groups increased their understanding of others’ roles and communication strategies (66.6%–86.6%).
[A3]			
Ellis et al. (2022)	Perception of IPE experience	2 questions for PD medication safety, correct percentage	•All groups significantly improved their understanding of the inappropriateness of withholding medication from a patient with PD ( *p* < .001). (Pre: 62.4%/Post:96.0%)
USA	Medication knowledge	2 questions for self-perceived comfort and competency, range 0-5	•All group significantly improved their understanding of the administration of PD medication (*p* < .001). (Pre: 79.2%/Post:92.1%)
[A4]			•All groups reported better comfortability scores after the IPE class (*p* < .001). (Pre: 3.01/Post: 3.74)
			•All groups reported better competency scores after the IPE class (*p* < .001). (Pre: 2.56/Post: 3.60)
Hardisty et al. (2014)	Perception of IPE experience (RIPLS)	RIPLS	•Quantitative results are not reported in this article.
UK		- 12 items, 5-point Likert scale	•Only qualitative results
[A5]		*Open-ended questions for satisfaction and lessons learned	
Kayyali et al. (2019)	Perception of IPE experience (perceived impact and assessment of IPE class)	20 items, 5-point Likert scale	•Most students answered felt the IPE class enhanced their communication and nursing & pharmacy skills (93.0%).
UK		- 12 items about session components and perceived impact, 4 tick box questions, 4 free text responses	
[A6]		* Open-ended questions for views on the scenarios, most enjoyable aspects, skills developed, fitting IPE in the curriculum and thoughts on possible improvements	
Meyer et al. (2017)	Perception of IPE experience	ATHCTS	•Students answered that the IPE simulation increased their medication knowledge (91.6%).
USA	Medication knowledge	- 21 questions, 6-point Likert scale	•Students answered that the IPE simulation taught them how to apply pharmacology to patient cases (90.2%).
[A7]		5 items, 5-point Likert scale	
		*Open-ended questions for perceptions of the impact of the simulation on their pharmacology knowledge andinterprofessionalism	
Motycka et al. (2018)	Perception of IPE experience (team structure, leadership, situation monitoring, mutual support, communication)	T-TAQ	•Team structure (Pre: 4.5/Post: 4.7) (*p* < .001)
USA		- 30 items, 6-point Likert scale	•Leadership (Pre: 4.7/Post: 4.8) (*p* < .005)
[A8]			•Situation monitoring (Pre: 4.5/Post: 4.8) (*p* < .001)
			•Mutual support (Pre:3.0/Post: 3.1) (*p* < .011)
			•Communication (Pre:4.0/Post: 4.2) (*p* < .001)
Paterson et al. (2015)	Perception of IPE experience (RIPLS)	RIPLS	•RIPLS score (Pre: 55.1/Post: 59.8) (*p* = .019)
UK		- 12 items, 5-point Likert scale	•Self-efficacy score (Pre: 126.9/Post: 144.9) (*p* = .010)
[A9]		Self-efficacy score	
		- 16 items, range 16 – 160	
		*Open-ended questions for satisfaction	
Powell et al. (2020)	Medication knowledge	Pre-post knowledge-based quiz score	•Pulmonary medication (Pre: 6.8/Post: 9.3) (*p* < .001)
USA			•Antibiotics (Pre: 4.2/Post: 7.8) (*p* < .001)
[A10]			•Cardiovascular medication (Pre: 4.6/Post: 8.6) (*p* < .001)
			•Diabetes medication (Pre: 5.4/Post: 9.75) (*p* < .001)
			•Antidepressant & antipsychotics (Pre: 5.0/Post: 8.1) (*p* < .001)
			•Pain management (Pre: 7.4/Post: 10.3) (*p* < .001)
			•GERD & inflammatory bowel disease (Pre: 4.6/Post: 7.6) (*p* < .001)
Ragucci et al. (2016)	Perception of IPE experience (satisfaction with IPE, confidence, and comfort with disclosing medical errors)	ICSW	•Students answered that IPE improved their teamwork skills at 4.2 on Likert scale.
USA		- 9 questions, 5-point Likert scale (1=strongly disagree, 5=strongly agree)	•Disclosing medication errors (Control: 67%/ Intervention: 97%) (*p* < .05)
[A11]			
Schussel et al. (2019)	Perception of IPE experience (attitudes)	Pre-post IPE survey	•Perceived knowledge and skills (Pre: 6.7/Post: 7.0)
USA	Medication knowledge (perceived knowledge and skills)	- 2 themes, 7-point Likert scale	•Attitudes (Pre: 5.6/Post: 5.8)
[A12]		*Open-ended questions for students’ reactions to the program and perceptions regarding their experience	
Stewart et al. (2010)	Perception of IPE experience (RIPLS)	RIPLS	•Knowledge and Awareness (Pre: 53.9/Post: 69.8) (*p* < .001)
UK		- 19 items, 5-point Likert scale	•Shared learning (Pre: 67.9/Post: 76.6) (*p* < .001)
[A13]			•Communication and team working (Pre: 81.4/Post: 82.5) (*p* < .001)
Young et al. (2021)	Perception of IPE experience (satisfaction, learning experience, perceived clinical application)	SPICE-R2	•Students strongly agreed that the workshop program promoted communication across professions for medication safety.
Australia		- 5-point Likert score	•There was strong agreement that working with students from different professions enhanced their education and their ability to work on an interprofessional team.
[A14]		*Open-ended questions for interactions, recognitions, and lessons learned	

n/r = Not reported; IPE = Interprofessional education; AD = Activity delivery; AO = Activity outcome; PD = Parkinson’s disease; RIPLS = Readiness for interprofessional learning scale; ATHCTS = Attitudes toward health care team scale; T-TAQ = Teamwork attitudes questionnaire; GERD = Gastroesophageal reflux disease; ICSW = Student satisfaction with the interprofessional communication skills workshop; SPICE-R2 = Student perceptions of interprofessional clinical education-revised instrument, version 2.

## Data Availability

Not applicable.

## Appendix 1. List of studies included in scoping review

A1. Achike FI, Smith J, Leonard S, Williams J, Browning F, Glisson J. Advancing safe drug use through interprofessional learning (IPL): a pilot study. The Journal of Clinical Pharmacology. 2014;54(7):832-839. https://doi.org/10.1002/jcph.289

A2. Clay AS, Chudgar SM, Turner KM, Vaughn J, Knudsen NW, Farnan JM, et al. How prepared are medical and nursing students to identify common hazards in the intensive care unit? Annals of the American Thoracic Society. 2017;14(4):543-549. https://doi.org/10.1513/AnnalsATS.201610-773OC

A3. Curley LE, Jensen M, McNabb C, Ram S, Torrie J, Jowsey T, et al. Pharmacy students' perspectives on interprofessional learning in a simulated patient care ward environment. American Journal of Pharmaceutical Education. 2019;83(6):6848. https://doi.org/10.5688/ajpe6848

A4. Ellis DM, Hickey S, Prieto P, McLaughlin C, Felgoise SH, Becker M, et al. Parkinson's disease medication administration during a care transition: the impact of interprofessional team simulation on student competency, comfort, and knowledge. Nursing Education Perspectives. 2022;43(3):164-170. https://doi.org/10.1097/01.NEP.0000000000000920

A5. Hardisty J, Scott L, Chandler S, Pearson P, Powell S. Interprofessional learning for medication safety. The Clinical Teacher. 2014;11(4):290-296. https://doi.org/10.1111/tct.12148

A6. Kayyali R, Harrap N, Albayaty A, Savickas V, Hammell J, Hyatt F, et al. Simulation in pharmacy education to enhance interprofessional education. International Journal of Pharmacy Practice. 2019;27(3):295-302. https://doi.org/10.1111/ijpp.12499

A7. Meyer BA, Seefeldt TM, Ngorsuraches S, Hendrickx LD, Lubeck PM, Farver DK, et al. Interprofessional education in pharmacology using high-fidelity simulation. Currents in Pharmacy Teaching and Learning. 2017;9(6):1055-1062. https://doi.org/10.1016/j.cptl.2017.07.015

A8. Motycka C, Egelund EF, Gannon J, Genuardi F, Gautam S, Stittsworth S, et al. Using interprofessional medication management simulations to impact student attitudes toward teamwork to prevent medication errors. Currents in Pharmacy Teaching and Learning. 2018;10(7):982-989. https://doi.org/10.1016/j.cptl.2018.04.010

A9. Paterson R, Rolfe A, Coll A, Kinnear M. Inter-professional prescribing masterclass for medical students and non-medical prescribing students (nurses and pharmacists): a pilot study. Scottish Medical Journal. 2015;60(4):202-207. https://doi.org/10.1177/0036933015606583

A10. Powell B, Jardine KD, Steed M, Adams J, Mason B. Enhanced nursing self-awareness and pharmacotherapy knowledge-base: peer-teaching and nursing/pharmacy interprofessional education. Medical Education Online. 2020;25(1):1814551. https://doi.org/10.1080/10872981.2020.1814551

A11. Ragucci KR, Kern DH, Shrader SP. Evaluation of interprofessional team disclosure of a medical error to a simulated patient. American Journal of Pharmaceutical Education. 2016;80(8):138. https://doi.org/10.5688/ajpe808138

A12. Schussel KE, Forbes S, Taylor AM, Cooley JH. Implementation of an interprofessional medication therapy management experience. American Journal of Pharmaceutical Education. 2019;83(3):6584. https://doi.org/10.5688/ajpe6584

A13. Stewart M, Kennedy N, Cuene-Grandidier H. Undergraduate interprofessional education using high-fidelity paediatric simulation. The Clinical Teacher. 2010;7(2):90-96. https://doi.org/10.1111/j.1743-498x.2010.00351.x

A14. Young J, Zolio L, Brock T, Harrison J, Hodgkinson M, Kumar A, et al. Interprofessional learning about medication safety. The Clinical Teacher. 2021;18(6):656-661. https://doi.org/10.1111/tct.13430
